# Heat Capacity and Thermodynamic Properties of Poly(chlorotrifluoroethylene) from 2.5 to 620 K

**DOI:** 10.6028/jres.097.014

**Published:** 1992

**Authors:** Shu-Sing Chang, James J. Weeks

**Affiliations:** National Institute of Standards and Technology, Gaithersburg, MD 20899

**Keywords:** automated adiabatic calorimetry, crystallite distribution, differential scanning calorimetry, glass transition, heat capacity, poly(chlorotrifluoroethylene), residual entropy, thermodynamic properties

## Abstract

Heat capacities and thermodynamic properties of a number of poly(chlorotrifluoToethylene) samples subjected to various thermal treatments, to achieve crystallinities ranging from approximately 10 to 90%, have been studied from 2.5 to 370 K by automated adiabatic calorimetiy and from 250 to 620 K by differential scanning calorimetry. Small heat capacity discontinuities in the temperature range from 320 to 350 K were observed in all samples with crystallinities greater than 40%. Spontaneous adiabatic temperature drifts associated with these anomalies were prasitive (exothermic) for quenched samples and negative (endothermic) for annealed samples. Therefore these anomalies were believed to be associated with a relaxation phenomenon similar to that of a glass transition. For highly quenched low crystallinity films, a much larger heat capacity discontinuity of greater than 15% was observed, amidst a crystallization exotherm. In addition to the above phenomena, annealing of the sample at any temperature between 240 to 400 K would produce a shift in the population distribution of crystallites from reorganization or melting and recrystallization. As a result, the apparent heat capacity became somewhat lowered at the annealing temperature and somewhat raised at about 20 K above the annealing temperature.

## 1. Introduction

Although poly(chlorotrifluoroethylene), PCTFE, is a fairly well-studied polymer in its physical and mechanical properties, its heat capacity and associated thermodynamic properties are only reported in a few publications [1-4] covering the temperature ranges: 2.5 to 20 K [2,4], 80 to 340 K [3] and 273 to 515 K [1]. There are also thermal analytical studies concerned mainly with the melting and crystallization phenomena near the fusion region. Glass transition phenomena were not observed by thermal means in the above mentioned publications. Dilatometric studies [5,6], however, have indicated the occurrence of a glass transition near 325 K. Dielectric [7,8] and mechanical relaxation [9] studies have also indicated a glass transition-like relaxation together with crystalline relaxations.

Since the crystallinity of PCTFE can be changed over a wide range [6], the heat capacity behavior of three PCTFE samples of varied crystallinity and thermal treatments was studied in this work with a fully automated, high precision, adiabatic calorimeter [10], for the temperature range from 2.5 to 370 K, and by a commercial differential scanning calorimeter from 250 to 620 K. The precision of the adiabatic calorimeter, except at temperatures below 50 K, was of the order of 0.02%. The high sensitivity in the detection of spontaneous temperature changes, on the order of 10^−5^ K, enabled the detection of the occurrence of broad relaxation phenomena, such as that occurring in polyethylene [11]. Techniques used in the adiabatic calorimetry enabled the separation of the underlying heat capacity contributed by lattice vibrations from that contributed by spontaneous thermal events occurring in the sample. The present work confirmed the existence of a glass-like relaxation in PCTFE, occurring in the temperature region from 320 to 350 K. In the transition region, the spontaneous adiabatic temperature drifts were positive (exothermic) for quenched samples and negative (endothermic) for annealed samples.

Data from differential scanning calorimetry, covering a temperature range from 250 to 620 K, were used to supplement the adiabatic calorimetry at temperatures above 370 K. The fast thermal analysis operation enabled the study of a large number of samples with various thermal histories. In addition to the confirmation of the existence of the glass transition discontinuities in most samples studied, crystallization and melting were also studied. The glass transition occurs amidst a complicated crystalline reorganization phenomenon over a wide temperature range from 240 to 400 K. By following rigorous calibration procedures, heat capacities and heats of fusion were obtained with reasonable precision using scanning calorimetry.

Combining the results from both adiabatic and scanning calorimetry, thermodynamic functions of the crystalline and the amorphous phases may be estimated. The amorphous phase contains a residual entropy at 0 K.

## 2. Experimental Detail

### 2.1 Materials

The adiabatic calorimetric samples of poly(chlorotrifluoroethylene), PCTFE, were composed of typical factory production KEL-F 81 brand plastics, KF-6060 pellet and KF-6061 powder, from Minnesota Mining and Manufacturing Company.^2^ Both batches were of Grade 3 quality with the degree of polymerization of about 1600, or a molecular weight of near 190,000. The molecular weight of the repeating monomeric unit, –CClF–CF_2_–, is 116.47 g mol^−1^ Three samples were loaded into the adiabatic calorimeter: (1) KF-6060 pellets as received, (2) high crystallinity sample prepared by slow crystallization from molten KF-6061 powder, and (3) quenched films made from KF-6061 powder. Table 1 lists loading conditions for the three adiabatic calorimetric samples, along with their initial densities *ρ*_i_; and crystallinities *X_i_.* After the evacuation, a small amount of helium gas at a room temperature of about 296 K was sealed with the sample to aid the thermal conductivity. The amount of helium gas was equal to the weight of helium used when the empty sample container was measured.

In order to achieve a maximum degree of crystallinity, a slow melt-crystallized sample was prepared by heating KF-6061 powder in vacuum above the melting point to 525-535 K, until all bubbles collapsed into a clear liquid. A motorized voltage source was used to slowly decrease the power to the heating mantle. The melt was cooled at a rate of 1-2 K per d, from 500 to about 400 K.

To achieve the lowest degree of crystallinity, thin films of PCTFE of about 0.15 mm thickness were prepared by pressing the melt of KF-6061 powder between two aluminum plates at about 575-590 K. The plates and the molten film were then quenched in icy water. In most of the films, the center portion of the film showed effects of air pockets and some anisotropic characteristics as the samples were viewed between crossed polarizers. Although the ranges of the densities of the clear region and the anisotropic region were about the same at 2.095±0.005 g cm^−3^ in order for the calorimetric sample to retain as high an amorphous character as possible, the optically anisotropic regions were cut off and not used.

Initial crystallinities of the three samples were estimated from their densities as 0.13, 0.54, and 0.87 for the film, pellet, and melt-ciystallized samples, respectively. The densities [6] of 2.076 and 2.186 g cm^−3^ for the amorphous and the crystalline phases, respectively, were used for the crystallinity estimation. For DSC measurements, besides the samples described above, the KF-6061 powder as received, quenched films made from KF-6060 pellets, a manufactured PCTFE tubing of 0.11 mm thickness, and a low molecular weight PCTFE KEL-F 200 Wax were also used.

If the quenched films had not been exposed to 325 K or higher temperatures, the x-ray *(CnKα)* diffraction patterns of these highly amorphous samples all showed an intense halo centered around a *26* value of 15.5° or around a spacing of 5.7 Å plus two fainter broad halos at around 32° and 39° or around 2.8 and 2.3 Å, similar to that found in commercial PCTFE films [12,13]. A faint halo at 8° or about 11 Å was always observed. For crystalline PCTFE, regardless of thermal treatments and degrees of crystallinity, the most intense lines are located at 5.5, 5.4, 5.2, and 4.9 Å, which are followed by two groups of fainter rings starting at 3.2 and 2.8 Å, as observed previously [14]. However, in all crystalline samples, there appeared also the halos at 11 and 2.3 Å. In addition, there was also a diffused ring at 14.8° or 6.0 Å.

The x-ray pattern of the low molecular weight KEL-F 200 wax, above its melting point at 373 K, appeared just like the amorphous film. At 409 K, the major halo shifted to 15° or the spacing increased to 5.9 Å. At room temperature, crystallized wax showed rings similar to that of the crystalline high molecular weight PCTFE, except the rings are more diffuse and somewhat difficult to resolve.

### 2.2 Adiabatic Calorimetry

A fully automated vacuum adiabatic calorimeter operating in the temperature range from 2 to 370 K [10] was used in this investigation. The calorimetric system included an automated analog adiabatic shield control system as improved from an earlier description [15], and a digital system for data acquisition and experimental control [10]. The analog control system was capable of maintaining the shield temperature and following the temperature changes of the sample container to within 1 mK. The principal measuring instruments in the digital system included a high resolution, self-balancing potentiometer [16] as modified from a commercially available manual potentiometer of a Diesselhorst ring [17] design, highly stable constant current sources, and a commercially available nanovolt amplifier with a linearity of 0.01%. This system was capable of measuring 0,1 V at full-scale with a resolution of less than 10 nV. Therefore this system provided a temperature resolution of near 10^−5^ K at temperatures above 50 K, from a nominal 25 Ω, platinum resistance thermometer operating at a current of 1-2 mA.

Above 14 K, the temperature scale used was IPTS-1968 [18]. At lower temperatures, the platinum resistance thermometer was calibrated against a germanium thermometer which was in turn calibrated against the NBS provisional temperature scale 2-20 (1965) [19]. The temperature scale below 14 K may either be interpolated directly from the calibration data or by following a three point calibration procedure [20]. At temperatures below 273 K, the scale IPTS-1968 differs from ITS-90 [21] by a maximum of 14 mK in the temperature region of 130 to 160 K. Between 273 and 373 K, the size of the temperature unit changes by 0.025%. The greatest change of 0.1% in the size of the temperature unit occurs at temperature regions of between 15-20 K and 50-60 K. The changes in the size of the temperature unit directly affects the heat capacity value calculation. Since the changes were 0.1% or less, no corrections were made to change the temperature scale from IPTS-1968 to IPS-90.

The heat capacity, *C_p_*, of the sample container assembly, including the sample, the container, the thermometer and the heater, was determined by the equilibrium temperature rise produced by an electrical energy input. The decay time constant toward the establishment of a temperature equilibrium after a disturbance was in the order of 100 s for this assembly [22]. Approximately 15 min were required after each energy input to reestablish a temperature equilibrium, or to reach a temperature drift on the order of or less than 0,1 mK min^−1^. The small drift arises from heat exchanges between the calorimeter and its surroundings due to a residual deviation in the control of a strict adiabatic condition. The time constant is much shorter at low temperatures due to the increase in the thermal conductivity of most materials. Much longer decay time constants with temperature drifts greater than that expected from the non-adiabatic conditions of the shield control system indicated the presence of a spontaneous thermal effect originating in the sample. Even under these adverse conditions, the instantaneous or short term heat capacities, contributed mainly from lattice vibrations, could still be estimated by extrapolating the drifts to obtain the temperature rise at the middle of an energy input. The energy of the spontaneous thermal event over a temperature range is obtainable by summing all energy inputs under adiabatic conditions and then subtracting from it the enthalpy increments as integrated from the lattice vibration heat capacity curve.

### 2.3 Differential Scanning Calorimetry

Some of the early heats of fusion and melting points were observed with a Perkin-Elmer DSC-IB differential scanning calorimeter. Most of the thermal analyses were performed with a Perkin-Elmer DSC-2 differential scanning calorimeter. The accuracy of the DSC measurements depended strongly on calibrations and the correctness of the assigned baseline for each scan. Meltings of indium and benzoic acid were used to calibrate the scales for the temperature and the power. Although scans of different rates were sometimes used, most of the runs were performed at a heating rate of 10 K min^−1^. Due to the thermal conductivity of the system, the temperature of the sample capsule lags behind the temperature of the thermometer/sample holder assembly as a function of heating rates by about 0.06 K (K min^−1^)^−1^ For better precision in heat capacity determinations, calibration scans of sapphire discs were frequently performed and the results compared against standard values [23,24]. For even better precision in heat capacity determinations, short scans with scanning temperature ranges of 5 to 10 K were used. Under these conditions, the uncertainty in the baseline is minimized. The average heat capacity for the 5 to 10 K interval may also be calculated by integrating the scan curve for the enthalpy and dividing the latter by the temperature interval, as in the intermittent heating method used in the adiabatic calorimetry. Heat capacities obtained by the short range method were found in agreement with adiabatic values to better than 0.5%.

Although DSC scans were performed at much lower temperatures, only the data above 250 K, where *C_p_* of PCTFE began to vary significantly with crystallinity, were used to supplement the adiabatic data.

One peculiar result was observed when heat capacities were calculated from continuous scans at temperatures above 500 K. The heat capacity values from continuous scans became progressively higher than that from short scans by about 20% at 600 K. Similar results were observed when the heat capacities of copper and teflon were measured. The reason for the occurrence of this discrepancy is unknown. It may be of instrumental nature or due to contamination or chemical reactions. Therefore long scans at high temperatures were used only for the observation of the melting points and the heat of fusion. The short scans were used for the heat capacity of the liquid at high temperatures.

## 3. Results and Discussion

### 3.1 Adiabatic Calorimetry

**Fig. 1:**
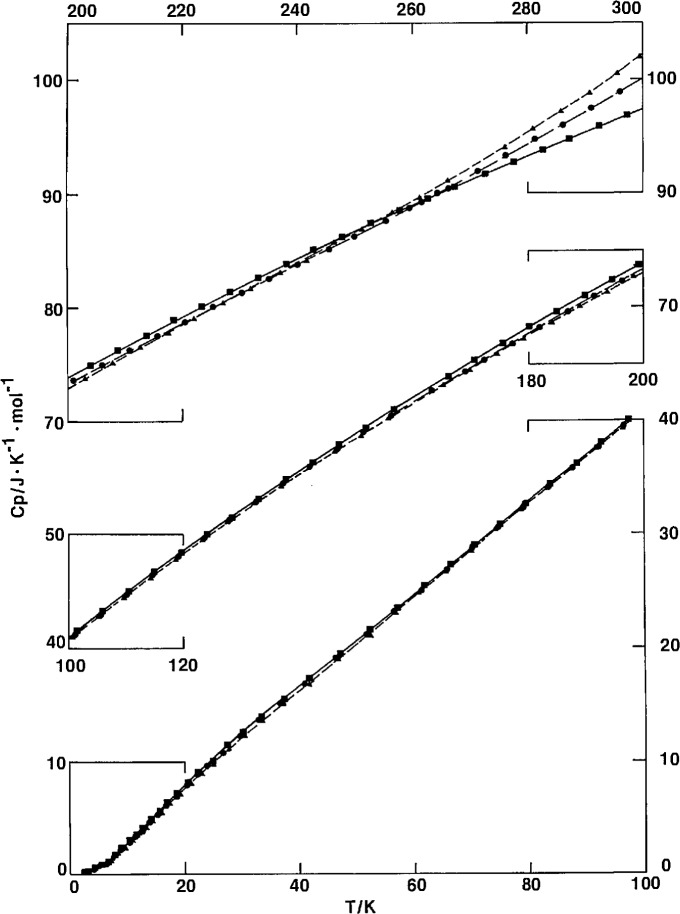
Heat capacity of PCTFE. Film: ▲. Pellet: ●. Slow crystallized: ■.

**Fig. 2:**
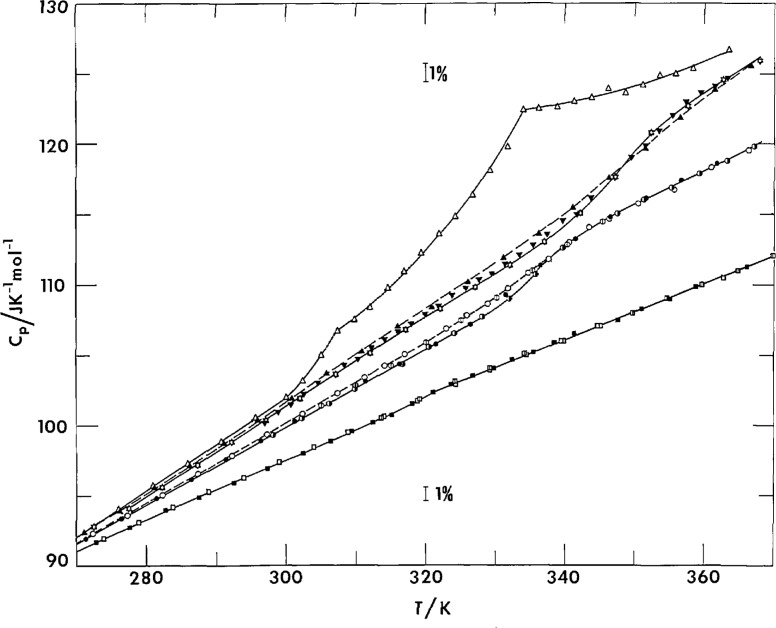
Heat capacity of PCTFE and glass transition. Film: △–F3, ▲–FQ, ▼–FSC, ✡–FRT. Peliet: ○–Pl, ⏀–PQ3, ◑–PA, ●–PSC. Stow crystallized: □–X1, ◫–XQ1, ■–XQ2.

The conditions and thermal histories before each series of adiabatic calorimetric measurements on the three samples are listed chronologically in Table 2. The results of the heat capacity measurements are listed in Table 3 and shown in different regions in Figs. 1 and 2. For clarity, not all series of measurements are shown in Fig. 1, as the results are close to each other below 250 K. As mentioned previously, the heat capacity reported here from adiabatic calorimetry represents mainly the vibrational contribution, free from the influence of long term spontaneous drifts due to crystallization and glassy relaxation.

#### 3.1.1 Heat Capacity

**Fig. 3:**
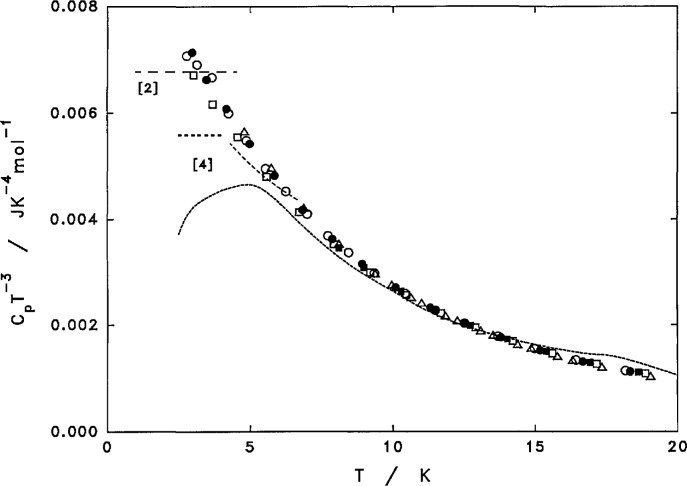
Low temperature heat capacity of PCTFE. Film: Δ–F1, F3. Pellet: ○–PQ2, ●–PA. Slow crystallized: □–XQ2, ■–XSC. Long dashes-Ref. [2]. Short dashes-Ref. [4].

At low temperatures, a Debye *T*^3^ region was not observed in this investigation. The values of *C_p_/T*^3^ were still increasing rapidly for all samples even at 2.5 K as the temperature was lowered, see Fig. 3. This is characteristic of the presence of a large amount of disorder and soft mode of vibration in all PCTFE samples, regardless of their crystallinities as estimated by the density. The rise of *Cp/T*^3^ to a peak, before a possible Debye region occurs at a lower temperature, has been observed generally in the temperature range of 2-5 K for almost all glassy materials. This peak occurs at 4 K for *cis*-1,4-polyisoprene [25], 3 K for amorphous selenium [26,27], 3-7 K for a number of thermoset resins as summarized in Ref. [28], and 3 K for polystyrene [29–31]. Similar to this investigation, the *Cp/T*^3^ values have not yet reached a peak even at 2 K for glassy *o*-terphenyl [32] and poly(vinylchloride) [33]. Weaker peaks also exist in highly crystalline polyethylene [34], crystalline *o*- and *p*-terphenyl [32,35] and even in a hard crystalline material such as sapphire [23].

There are two publications [2,4] reporting the cryogenic behavior of PCTFE. From thermal conductivity measurements of a sample of PCTFE with a density of 2.114 g cm^−3^ from 1 to 4.5 K [2], an average value for *C/T^3^* of 0.123 mJ K^−4^ cm^−3^ or about 6.8 mJ K^−4^ mol^−1^ was given. This value is approximately the average of the values observed in the present study, see Fig. 3.

The results of specific heat measurements for a sample of PCTFE without detailed description from 2.5 to 20 K are reported in several ways [4], which are also shown in Fig. 3 for comparison. The graphical data were estimated from a log(*C*)-log(*T*) plot for *T*<10 K and from a *C/T-T*^4^ plot for *T*>10 K from their publication. The data at 20 K was also obtained from the log-log plot, as the *T*^2^ plot only extended to 18 K. Only in the region between 6 to 14 K, do the graphical data agree reasonably well with our data. Below 5 K, the graphical data indicate a lowering of *C/T*^3^ from 4.6 mJ K^−4^ mol^−1^ at 5 K to 3.7 mJ K^−4^ mol^−4^ at 2.5 K. The value at 2.5 K was nearly one-half of that from this study and that from a thermal conductivity study [2]. The authors, however, assigned a much higher Debye constant of 5.6 mJ K^−4^ mol^−1^, which is much higher than their graphical data, but is still lower than that in the present study. The authors also assigned a constant for a *C/T*^2.5^ dependency for the range 4.3 to 6.7 K, which yielded values much closer to the values of the present study. The reasons for the different representations and the discrepancies among their graphical data and assigned constants are unknown.

It may also be noted that a mixture of PCTFE grease in silica gel was reported [36] to have heat capacities almost five times higher than the values reported by us and in Ref. [2] for PCTFE alone, in the temperature range from 1.8 to 4.5 K.

**Fig. 4:**
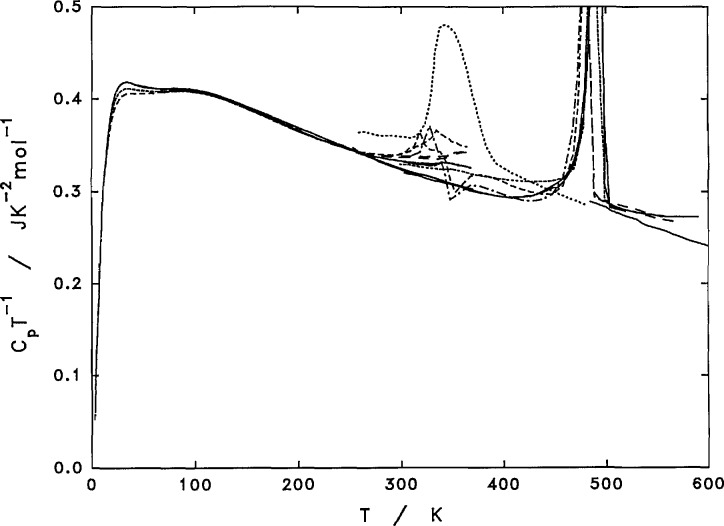
Heat capacity function of PCTFE (adiabatic calorimetry and DSC). Solid lines–slow crystallized. Short dashes–pellet. Medium dashes-film (F1–F3). Long dashes-film (FAQ, FAS, FRT). Dots–wax.

A region of *C_p_* proportionality to *T* exists between 30 and 100 K, as seen in Figs, 1 and 4. The heat capacity function, *C_p_/T*, curves in Fig. 4 also included a few DSC scans as discussed in later sections. Above 100 K, *C_p_*, of PCTFE still assumes a relatively linear dependency on temperature, as seen by slowly varying *Cp/T* values. The linear dependency and proportionality may be a result of a relatively uniform distribution of vibrational frequencies from 100 to 1000 cm^−1^, as estimated by Guttman [37].

From 100 to 260 K, heat capacities of all samples studied are surprisingly close to each other, within a spread of about 1%, regardless of a large variation in sample densities or crystallinities. The spread is greatest, about 3%, around 30 K. However, over the wide temperature region from 10 to 250 K, the heat capacity is higher for samples of higher crystallinity. Generally the *C_p_* of most glassy materials is higher than the *C_p_* of their crystalline counterpart. Although examples have been cited [38] to show that the *C_p_* of the crystalline state is higher than that of the vitreous state in certain ranges of temperature, the occurrence of this phenomenon over such a wide temperature range is rather unusual.

Above 260 K, the heat capacity depends strongly on the ciystallinity or the complementary amorphous content. The lower the amorphous content, the lower the heat capacity, as normally expected of most semicrystalline materials.

Heat capacities of PCTFE above the cryogenic temperatures were only reported in two publications [1,3]. *C_p_* values between 80 and 340 K of two PCTFE samples with crystallinities of 0.46 and 0.65, as determined by densities, were studied and smoothed data on the high density sample reported [3], The reported values agree very well with that for our pellet sample series PI and PQ. Similar to our findings, the authors found that the heat capacities for the two samples were the same within their experimental error of 0.3% at temperatures below 325 K. Their highest temperature measurement stops in the glass transition region, just as the transition is about to be completed. The authors failed to detect or report a glass transition, except to note an indication of a positive d^2^*C_p_*/d*T*^2^. At 340 K, the heat capacity of the low density sample was about 0.8% higher than that of the denser sample.

Two samples of PCTFE, with crystallinities of 0.35 and 0.82 as determined from the heat of fusion, were studied with a differential calorimeter in a continuous heating mode for the temperature region from 0 to 250 *°*C [1]. No glass transition was reported for either of the two samples. The results from these two samples will be compared with DSC studies of the present work in later sections of this paper.

#### 3.1.2 Glass Transition and Tacticity

**Fig. 5:**
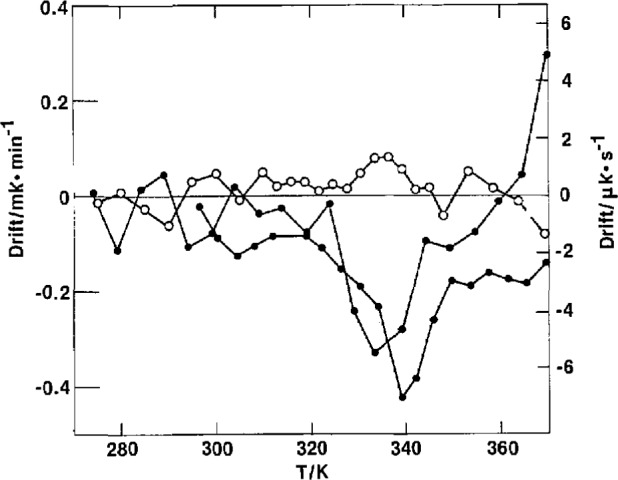
Adiabatic temperature drift of pellet. ○–quenched. ●–annealed.

A glass-like transition was observed in all adiabatic calorimetric samples in the region from 320 to 350 K, as shown in Fig. 2. The intensity, as viewed by the magnitude of the sudden heat capacity rise, Δ*C_p_*, is a function of the amorphous content. In the transition region, the spontaneous adiabatic temperature drifts were positive (exothermic) for quenched samples and negative (endothermic) for annealed samples. Although there appeared a small *Cp* discontinuity at just below 320 K for the highly crystalline sample X, the drifts were all within 1 μK s^−1^ after 15-20 min following the termination of energy input. Figure 5 shows the spontaneous drifts for the pellet sample P, with the annealing peak occurring at a higher temperature than the quenched peak.

**Fig. 6:**
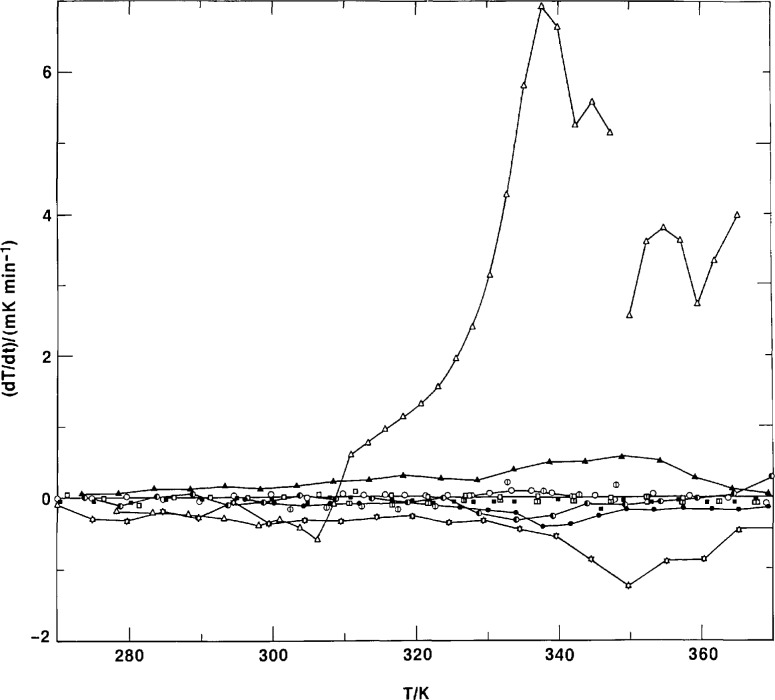
Adiabatic temperature drift of film. Film: Δ–F3, ▲–FQ, ▼–FSC, ✡–FRT. Pellet: ○–P1, ⏀–PQ3, ◑–PA, ●–PSC. Slow crystallized: □–X1, ◫–XQ1, ■–XQ2.

Figure 6 shows the drift behavior for the film sample F. For the first heating of highly amorphous films, there appeared an endothermic dip in the drift at about 307 K. This may be a result of being left standing at the room temperature of 296 K for some time, after the films were quenched from the melt in ice water. The true drift peak for the glass transition probably was masked by the larger exothermic drifts due to crystallization occurring at temperatures above 310 K. Subsequent to the crystallization and stabilization of the films for a long time at 370 K, normal drifts for the glass transition phenomenon appeared in the temperature range of 340-350 K, see Fig. 6. The heat capacity behavior of the film after the stabilization process lay in between that of the pellet and the first heating of the films, see Fig. 2.

The variation of the glass transition of PCTFE as a function of crystallinity contradicts the commonly perceived idea that the glass transition temperature increases with crystallinity. Except for the highly amorphous quenched film, where the glass transition may be very wide and the true center of the glass transition was not observed, *T_g_* appears to be lower for lower amorphous content. One plausible explanation for this phenomenon is that in the highly crystalline sample X, the remaining 10% (approximately) of amorphous content consists of mainly uncrystallizable low tactic or atactic portions of the polymer, and therefore the glass transition temperature appears near the low end of the glass transition range, as observed in the highly amorphous quenched film F.

**Fig. 7:**
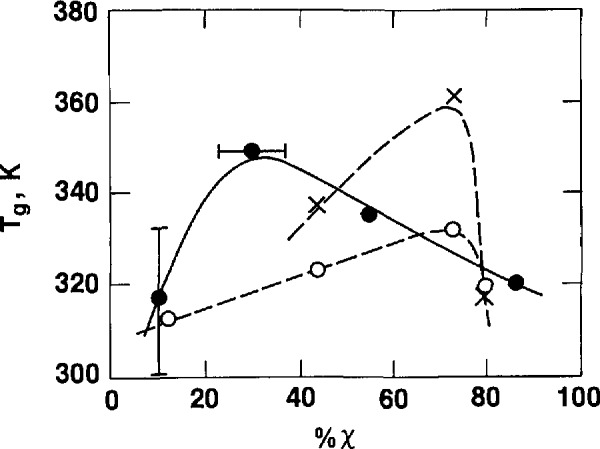
Glass transition temperature as a function of crystallinity. ●–this work. ○–dilatometrie [7,36]. ×–dielectric [7].

The variation of the glass transition temperature as a function of crystallinity is shown in Fig. 7, where results from dilatometric [7,39] and dielectric [7] studies are also included. Earlier dilatometric Studies [6] of samples with densities of 2.116 and 2.165 g cm^−3^ suggested a glass transition temperature of 325 K which was relatively invariant with crystallinity.

### 3.2 Differential Scanning Calorimetry

About fifty DSC samples were taken from PCTFE samples of the same batches as the samples for adiabatic calorimetry, plus the original powder, films made from pellets, a manufactured tubing, and a low molecular weight wax. These DSC samples were subjected to various thermal treatments to show the dependency on the thermal history, such as annealing, quenching, and slow cooling. The influence of mechanical deformation was also studied. It is not feasible to show all the results of some 300 DSC runs in any detail except to make a summary here. A few of the typical runs, especially the first heatings of quenched samples, are shown as heat capacity functions in Fig. 4, as well as heat capacity curves in Fig. 11 in a later section. Except for the low molecular weight wax, the results on all other PCTFE samples, whether they were composed of pellet, powder or manufactured tubing, showed nearly identical behaviors as dictated by the thermal history after melting.

The low molecular weight Kel-F 200 Wax, showed a broad melting region from 305 to 400 K, with a peak at 350 K and a heat of fusion of 15 ± 1 J g^−1^. The units of per gram instead of per mole are used here for convenience. The liquid heat capacity appeared to connect reasonably well (within 1%) to the adiabatic data for quenched film above its *T_g_* and is slightly lower than the DSC liquid heat capacity above the *T_m_* of the high molecular weight PCTFE. Upon cooling, crystallization starts around 375 K with a exothermic maximum at about 352-355 K. The wax showed some instability at temperatures above 510 K.

The following summarizes the DSC observations on various forms of pellet, powder and film samples with different thermal histories. The observations included glass transition, crystallization, crystalline reorganization and fusion.

#### 3.2.1 Glass Transition Temperature

Except for the highly crystalline sample X where the small Δ*C_p_* is difficult to observe by DSC, glass transition temperatures as depicted by a *C_p_* discontinuity were also observed by DSC in the temperature range of 330-360 K, in similar magnitudes of Δ*C_p_* as that observed by adiabatic calorimetry. The observation on the sample P was shown in Fig. 4 as an example. The slight increase in the observed glass transition temperature is due to the kinetic nature of the glass transition and the dynamic nature of the DSC measurement.

In order to improve the thermal conductivity and thus the consistency of sample temperature with the indicated DSC temperature, film samples were scanned as they were immersed in silicone oil. The *T_g_* of films held overnight at 370 K was found to be in the range of 355 to 360 K. Only if the cooling rate was below 0.1 K/min or if the sample was annealed at 340 K for some time, did the transition range became narrower with a small relaxation peak at 360 to 375 K. Samples cooled at higher rates show a broader transition range of about 30 K.

For high amorphous content samples, apparent endothermic peaks occurred at about 320 K for the first heating of film sample F and films quenched from molten pellets KF-6060. A manufactured thin tubing of 0.11 mm thickness, left at room temperature for about 18 years, was scanned to reveal an even larger endothermic peak at 329 K, see Figs. 4 and 11. The onset of these endothermic peaks were the result of the rapid rise of *C_p_* in the glass transition region, as seen by the adiabatic calorimetry, where spontaneous effects and crystallization effects were largely eliminated to reveal the underlying instantaneous lattice heat capacities.

However the nature of these peaks was rather complicated. The complexity could arise from a variety of origins, such as the glassy relaxation peak, the crystalline reorganization peak as described in a later section, or the termination of the *C_p_* rise by the onset of the crystallization exotherm.

#### 3.2.2 Crystallization

Crystallization of quenched films observed at a scan rate of 10 K min^−1^ started near 335 K with an exothermic maximum at about 355 K, Figs. 4 and 11, regardless of whether the film was quenched from the melt to room temperature water, icy water, or an acetonedry ice mixture at 195 K. The crystallization exotherm for the films was on the order of 3 J g^−1^. Upon further heating, melting points for the first heating of quenched films were observed at 481–482,5 K with heats of fusion of 10.5–12.7 J g^−1^. The slower adiabatic calorimetry indicated the onset of crystallization for the quenched films near 310 K with a maximum rate near 340 K by the observations of adiabatic temperature drifts, Fig. 6.

A manufactured PCTFE tubing of 0.11 mm wall thickness, left at room temperature for 18 years, showed a crystallization onset at about 325 K and a maximum at about 350 K with a heat of crystallization of about 5 J g^−1^ during the first heating. Further heating produced a melting point at 484 K with a heat of fusion of 16.3 J g^−1^ After cooling at −10 K min^−1^ from the melt with a heat of crystallization of 19 J g^−1^, the melting point is increased to 488 K with a heat of fusion of 19.3 J g^−1^ Although during the first heating of the tubing, it indicated a greater extent of crystallization than the films that we prepared, after the melting, the tubing behaved rather similarly to the samples made from either the pellets or from the powder.

The heat of crystallization of highly quenched films was much less than the subsequent heat of fusion. Therefore these highly amorphous films and the thin tubing were not completely amorphous but initially contained a certain crystallinity.

A film made from the powder, heated to 633 K and then quenched in water at room temperature, was studied for any stress-induced crystallization. It however behaved similar to films without any mechanical treatment. A strip of the film was drawn to break at about 50% elongation at room temperature. The 6 mm strip formed a neck of about 4 mm in width. A DSC scan of the neck area indicated a relatively large peak at 311 K, followed by a crystallization exotherm with a maximum at 352 K. The crystallization continued at temperatures above 410 K. The sample was cooled at 440 K Upon reheating the sample, crystallization started again at 465 K. This sample also showed reorganizational peaks at 316 and 354 K, for annealing at room temperature and 335 K, respectively. The melting point of this sample was at 487 K. The untreated film from the same batch showed reorganizational peaks of 310 and 345 K for room temperature and 345 annealing, a crystallization exotherm maximum at 356 K and a melting point of 483 K.

#### 3.2.3 Reorganization of Crystallites

**Fig. 8:**
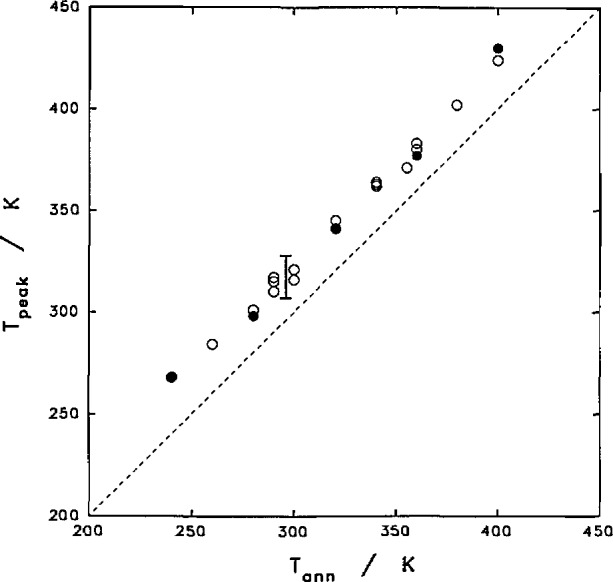
Reorganization peak temperature as a function of annealing temperature. ●–multiple peaks. ○–individual peaks. I—room temperature annealing.

The melting range for PCTFE is very wide, indicating a wide distribution of crystallite sizes and perfection. If a partially crystalline sample, obtained by a smooth continuous cooling procedure, was left standing at any temperature above 240 K for some time, the DSC thermogram would show a small dip at the annealing temperature and a small endothermic peak at about 20 K above the annealing temperature. Fig. 8. The dip in the DSC curve was not an exothermic phenomenon, but was caused by a slight depletion of the crystallite population that might be melting in the temperature region of concern. The small peak just above the annealing temperature was the result of the melting of the additional crystallite population from a previously existing smooth distribution. The small humps from annealing at temperatures above 400 K are more difficult to detect due to the superimposition onto the rapid rise of the thermogram of the main melting peak.

A more dramatic demonstration of this phenomenon was to create a sample with multiple small melting peaks. A film sample was first annealed at 400 K overnight and then cooled to lower temperatures at a rate of 10 K/min. The smooth cooling procedure was successively interrupted and the sample was annealed at 360,320, and 280 K for 2.5 h, and finally at 240 K overnight. Upon heating of this sample, small humps were observed at 268, 298, 341, 377, and 430 K, e.g., at about 20 to 30 K above the annealing temperatures. Figure 8 shows the results from this demonstration, as well as from many observations with a single annealing temperature. Non-smooth distributions of crystallite population in partially crystalline polymers with a wide (over 100 K) range of melting leading to multiple melting peaks have been observed in other polymers [40,41].

**Fig. 9:**
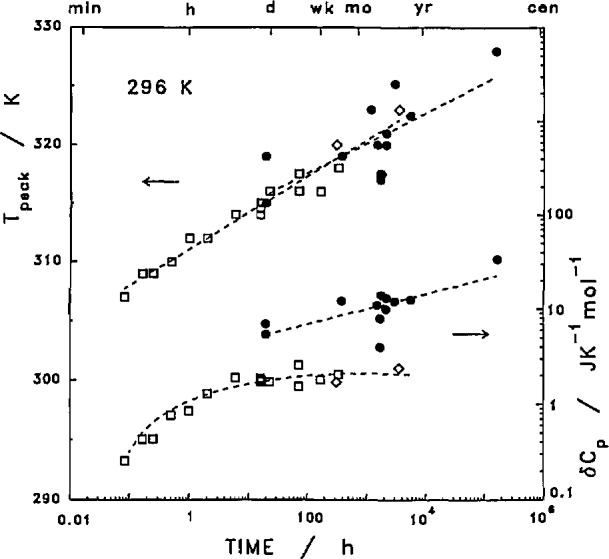
Reorganization peak temperature and intensity as functions of annealing time. □,◇–quenched films after crystallized at 400 K. ●–original films and tubing before heating to 400 K.

Both the position and the intensity of this reorganization peak were also functions of annealing time, Fig. 9. The reorganizational peak disappears if the sample is re-scanned immediately after a smooth cooling process. For samples crystallized at 400 K and then annealed at a room temperature of 296 K, the peak temperature varied from 307 to 320 K by annealing for a few minutes to several months. The peak height, as judged from the rescan, rose quickly with annealing time and then apparently leveled off after about 10 h at a level near 0.02 J K^−1^ g^−1^ or 2 J K^−1^ mol^−1^.

The increase of the peak temperature of the original film (including the manufactured tubing) appeared to have the same functionality as the increase of the peak temperature of the film after crystallization at 400 K. The commercially produced PCTFE tubing of 0.11 mm thickness yielded a peak at 328 K after standing at room temperature for 18 years. However, the growth of the much larger peak by annealing the originally quenched film at room temperature was rather different from that of the crystallized film. The peak height varied from 0.05 J K^−1^ g^−1^ for annealing overnight at room temperature and to about 0.2 J g^−1^ after room temperature storage for 18 yr. It is possible that some of the peak height is contributed by the relaxation phenomena of the glass transition.

#### 3.2.4 Melting Point and Heat of Fusion

The melting temperature of PCTFE was not well defined and the major portion of the melting occurred over a wide temperature region of over 50 K. The usual practice for the determination of a sharp melting point in power-compensated type of DSC, such as the instrument used here, is to estimate the onset temperature. The temperature difference between the peak and the onset for a sharp first order transition is a function of the sample size. However, the peak temperature for the broad melting of PCTFE was found to be relatively insensitive to the heating rate and the amount of sample used in the sample capsule. Therefore the peak temperature is chosen as the representative melting point, *T_m_*, for the sample.

For low crystallinity samples consisting of quenched films or manufactured tubing, additional crystallization occurred above 335 K as the samples were scanned for the first time to their melting points. Melting points and heats of fusion for the first heating of quenched film were observed at 481-482.5 K with heats of fusion of 10.5-12.7 J g^−1^ The tubing gave a *T_m_* of 484 K with Δ*H_m_* of 16.3 J g^−1^. As the energy release during the crystallization was about 3-5 J g^−1^, the original film or tubing before the additional crystallization would have yielded a heat of fusion of 7-11 J g^−1^.

Cooling the melt, regardless of the sample origin, at a rate of 5-40 K min^−1^ produces *T_m_* of around 486-489 K and a heat of fusion around 15-20 J g^−1^, similar to most of the pellet or powder PCTFE samples measured in as-received condition. Samples fast-cooled in DSC at an indicated rate of 320 K min^−1^ also yielded *T_m_* around 487 K, without the 315 K peak and the subsequent recrystallization at around 355 K. Thus the true cooling rate in the DSC was far less than that indicated.

**Fig. 10:**
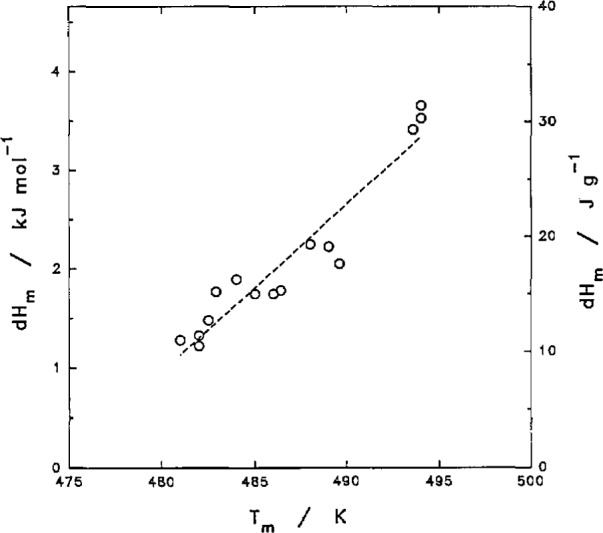
Heat of fusion and melting point of PCTFE.

For slow-crystallized samples, the melting points observed were about 494 ±1 K with a heat of fusion on the order of 29-30 J g^−1^ A sample held at 470 K overnight also gave a *T_m_* of 492 K. The heat of fusion from DSC, as estimated by integrating the peak area above a baseline drawn from the solidus heat capacity at about 430 K to the liquidus heat capacity at about 500 K, is a lower bound of the value. The results for the heat of fusion and melting point measurements for various PCTFE samples are shown in Fig. 10.

**Fig. 11:**
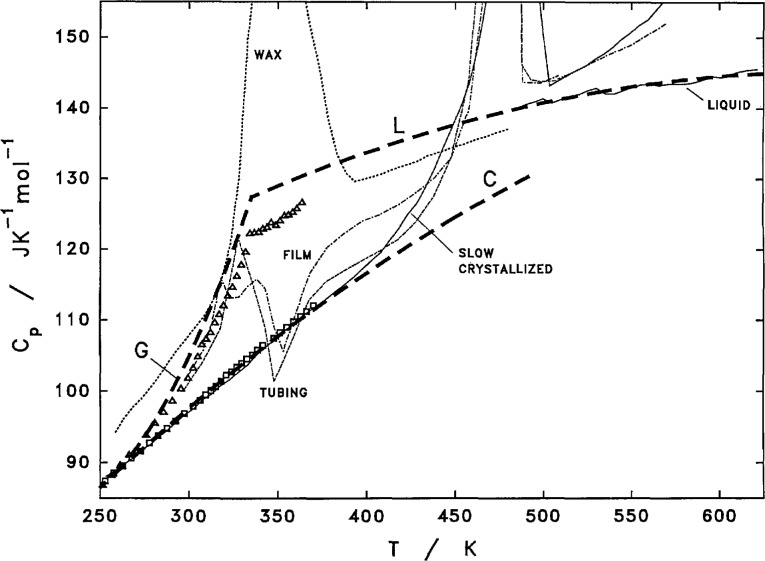
Estimated heat capacity behavior of PCTFE crystal, glass, and liquid. Heavy dashes–estimated heat capacity for PCTFE crystal (C), glass (G), and liquid (L). Adiabatic measurements: Δ–F3, □–XQ2. DSC measurements: wax, film (first heating), tubing (first heating), slow–crystallized and liquid (short scan) as labeled.

Based on the densities, the results of DSC measurements could only be extrapolated to about 35 J g^−1^ for the heat of fusion for the pure crystalline phase. A correction to the peak area, as based on the heat capacities of the crystalline and liquid phases as described in a later section and Fig. 11, would increase the heat of fusion value of the pure crystalline phase by 5 J g^−1^. The observed value for heat of fusion may be a lower bound for bulk crystals, due to the surface energy contributions from small crystallite sizes in these samples. We believe that a heat of fusion of about 40±5 J g^−1^ for the pure crystalline phase at an equilibrium melting point of 497 K [42] is perhaps a reasonable estimate.

Reference [1] reported heat of fusion values of 15.1 ±0.4 J g-i for air-quenched PCTFE and 35.1 ±0.8 J g^−1^ for a slow-cooled sample. The degrees of crystallinity for the two samples were estimated as 0.35 and 0.82, respectively, by using a value of 43.1 ±2.5 J g^−1^ as the heat of fusion of “pure” crystalline PCTFE from solubility studies [43]. Reference [1] also gave a value of 46.9 ±4.2 J g^−1^ as the heat of fusion for the pure crystals estimated by a method briefly described as volumetric [44]. A value of 75.7 J g^−1^ was reported for the heat of fusion by another solubility study [45], which we judged to be too high in comparison to all other studies.

Using the heat of fusion of the pure crystalline phase as the basis, the quenched film or the tubing would have a crystalline content of about 25% before any additional crystallization set in at temperatures above 350 K. These residual crystallinities were small crystallites which were not detected by optical means and only gave a broad halo in x-ray powder patterns.

### 3.3 Thermodynamic Properties

#### 3.3.1 Heat Capacity of PCTFE Ciystal, Glass, and Liquid

In the determination of the high tem- perature heat capacity of PCTFE by DSC, a peculiar behavior was found. At temperatures above 500 K, heat capacities estimated from continuous scanning runs as calibrated with similar runs with sapphire discs became progressively higher than that obtainable by the short interval (5-10 K) scan or integration. This peculiar behavior was observed not only with the current PCTFE samples but also on heat capacity estimations of samples of teflon and copper, when compared with literature values. All results indicated deviations between the continuous scan and short interval scan of less than 0.5% at 430 K that increased to about 5% at 550 K. The values measured by the short interval integration method on copper or teflon were however near 0.2% of the literature values throughout the entire temperature range. Therefore the short interval integration values are considered to be close to the true heat capacity values, and the long or continuous scans are to be used only for the estimation of heats of fusion and melting points. The reason for this discrepancy is unknown. It may be due to instrumental artifacts or due to the deterioration or contamination of the sample holder assembly, and/or chemical reactions at high temperatures.

The heat capacity of crystalline PCTFE below 300 K is considered to be the same as that measured for the highly crystalline sample X, Table 4. As the differences between the glassy and crystalline phases are rather small below 300 K, no adjustment is applied to adjust for the near 10% amorphous content. Above 300 K, *C_p_* of the crystalline phase is represented by a curve as shown in Fig. 11, which is a smooth extension of the heat capacity behavior below 300 K. There is a slight reduction in the *C_p_* corresponding to the contribution from approximately 10% amorphous phase to the Δ*C_p_* for the glass transition.

The heat capacity of amorphous PCTFE in the glassy state below 250 K is considered to be the same as the quenched film before heating to temperatures above the room temperature. As the differences between the glassy and crystalline phases are rather small below 300 K, no adjustment is applied for any crystalline content in the quenched films. Above 250 K and extending to the supercooled liquid state, the heat capacity of the amorphous PCTFE is obtained by adding 25% of the Δ*C_p_* to the measured heat capacity of the quenched films, as shown in Fig. 11. As based on DSC measurements of heats of fusion, the quenched films may originally contain a crystallinity of about 0.25, before additional crystallization starts at temperatures just above the glass transition temperature.

The liquidus heat capacity, from 335 K in the supercooled liquid state to 620 K in the melt, was obtained by combining the adiabatic measurement of the quenched film from 335 to 370 K adjusted for the Δ*C_p_* due to a residual crystalline content in the original film and the DSC measurements of molten PCTFE above 495 K using the short interval scan as mentioned earlier. The trend of the liquidus *C_p_* behavior of a low molecular weight wax as observed with DSC was used as a guide to the functionality between the two measurements. The assigned liquidus heat capacity is also shown in Fig. 11.

#### 3.3.2 Thermodynamic Functions and Residual Entropy

The thermodynamic functions for the crystalline PCTFE are listed in Table 4. The functions are calculated based on the estimated crystalline heat capacity as mentioned above, with the assumption that there is no residual entropy at 0 K for the crystalline phase. If there is any residual entropy for the crystal, the free energy term should be corrected by an amount equal to *TS*_0_.

The thermodynamic functions of the amorphous phase and the liquid are listed in Table 5. These functions are calculated based on the estimated amorphous and liquid heat capacity as mentioned above. The integration for the enthalpy and entropy are based on the zero-point enthalpy and entropy of the glass rather than the crystal. As the amorphous phase is expected to have a residual entropy at 0 K, the free energy is not calculated.

The heat of fusion of the crystalline state is considered to be about 40 J g^−1^ or 4.7 kJ mol^−1^ at 497 K. The corresponding entropy of fusion is 9.3 J K^−1^ mol^−1^. These values will yield (*H*_liq_-*H*_x_)_497_ of 44.0 kJ mol^−1^ and *S*_497,liq_, of 179.5 J K^−1^ mol^−1^ Then (*H*_gl_-*H*_x_)_0_ will be about 1.6 kJ mol^−1^ and the residual entropy for the amorphous PCTFE at 0 K, *S*_0,gl_, will be about 2.0 J K^−1^ mol^−1^ This *S*_0,gl_ value, although reasonable, is smaller than the usual observation mentioned for a collection of reliable residual entropies [26,32] of around (*R* In 2)/2 or 3 J K^−1^ per each chain atom or bead. The smaller residual entropy may be the result of the disorder existing in the crystalline PCTFE or a low estimation of the heat of fusion of the PCTFE crystal.

## 4. Conclusion

One advantage of adiabatic calorimetiy over DSC is the ability to detect the direction and to separate the effects of relatively long term spontaneous thermal events originating in the sample. The DSC measurement provides only a single overall heatflow thermogram as a function of time during a scan. The adiabatic calorimetiy allows the extraction of short term equilibrium heat capacities such as contributions from lattice vibrations from long term spontaneous thermal events such as relaxation and crystallization, by observing the adiabatic temperature drifts as a function of time. Magnitudes, directions, and locations of the spontaneous event may also be measured by adiabatic calorimetry. Differential scanning calorimetry is however more advantageous if large numbers of small-size samples need to be tested at a lower precision.

The lattice heat capacities of PCTFE samples of different crystallinities clearly show a glass transition in the region from 320 to 350 K, heretofore undetected by other thermal measurements. The spontaneous drifts observed in adiabatic calorimetry have also confirmed the glass-like relaxation phenomenon, which give rise to a positive temperature drift for quenched glasses and negative drift for annealed glasses.

The glass transition temperature is, however, lowered for samples of higher crystallinity. This may be caused by the rejection of the uncrystallizable portion of a polymer chain, which itself may have a lower glass transition temperature than the overall glass transition temperature as contributed by all different structures.

Although a few approximations were applied to derive the thermodynamic functions for PCTFE crystal, glass, and liquid, the small residual entropy of the glass may be the result of a disorder in the crystalline structure. Heat capacities of the highly crystalline sample behaved almost identically to that of a low crystallinity sample in the cryogenic temperature region down to 2.5 K, where a region for the Debye *T*^3^ law was not yet reached.

The melting range of PCTFE is very wide, with the majority of the melting occurring in a range of 60 K, For ordinary samples with a smooth cooling history and having a crystallinity around 0.5, annealing at any temperature above 240 K may cause a reorganization into a non-smooth distribution of crystallite sizes. By successively annealing at lower temperatures, a sample with more than five melting peaks was prepared.

## Figures and Tables

**Table 1:** Adiabatic calorimeter loading of PCTFE samples

Designation	Mass (vacuo) g	*ρ*_i_ g cm^−3^	*X*_i_	He kPa
X (Slow crystallized)	124.617	2.170	0.87	4.2
P (Pellet, as received)	145.147	2.133	0.54	5.3
F (Film)	63.396	2.095	0.13	3.3

**Table 2:** Conditions and thermal histories of PCTFE

Designation		Range		Conditions
Sample	Run	*T*_i_, K	*T*_f_, K	
P	PI	300	350	As received, No liquid nitrogen refrigeration
	PQ1	65	205	Cooled from 350 K at −5 K/min
	PQ2	3	125	Cooled from 350 K at −5 K/min
	PA	3	370	Annealed at 320 K and cooled at −5 K/h
	PQ3	200	370	Cooled from 370 K at −6 K/min
	PSC	300	370	Slow cooled from 370 K at −0.5 K/h
X	XI	310	370	First run as prepared
	XQ1	150	355	Cooled from 370 K at −5 K/min
	XSC	8	175	Slow cooled from 340 K at −0.33 K/h
	XQ2	3	370	Cooled from 370 K at −5 K/min
F	Fl	10	16	Run as prepared
	F2	190	215	Run as prepared
	F3	5	365	First run as prepared
	FQ	65	370	Cooled from 370 K at −5 K/min
	FSC	265	370	Slow cooled from 370 K at −0.5 K/h
	FRT	295	365	1 month at room temperature

**Table 3:** Heat capacities of PCTFE (1 mol = 116.47 g)

*T* K	*C*_*p*_ J K^−1^ mol^−1^	*T* K	*C*_*p*_ J K^−1^ mol^−1^	*T* K	*C*_*p*_ J K^−1^ mol^−1^

Pellet					
P1					
305.01	101.33	320.07	105.88	335.24	110.91
309.98	102.81	325.08	107.39	340.28	112.80
315.01	104.23	330.14	109.01	345.35	114.39

PQ1					
65.40	26.66	112.28	45.49	161.94	62.24
69.25	28.24	115.77	46.77	165.63	63.38
71.68	29.21	119.28	48.06	169.27	64.48
75.05	30.63	122.82	49.34	172.87	65.54
78.44	32.03	126.29	50.56	176.52	66.63
81.86	33.43	129.81	51.79	180.23	67.71
85.17	34.80	133.36	53.02	183.90	68.78
88.53	36.17	136.85	54.21	187.52	69.81
91.88	37.53	140.39	55.39	191.21	70.85
95.24	38.88	143.98	56.67	194.95	71.91
98.61	40.21	147.51	57.70	198.65	72.94
102.00	41.54	151.09	58.85	202.32	73.95
105.41	42.87	154.72	60.00		
108.83	44.19	158.31	61.12		

PQ2					
2.56	0.13	13.67	4.55	67.72	27.64
2.60	0.15	14.98	5.21	72.11	29.44
2.78	0.15	16.46	5.96	76.43	31.25
3.15	0.22	18.18	6.83	80.80	33.15
3.66	0.33	20.14	7.79	85.23	34.88
4.24	0.46	22.46	8.91	89.64	36.70
4.86	0.63	24.95	10.07	94.04	38.46
5.52	0.83	27.63	11.27	98.45	40.23
6.23	1.10	30.87	12.67	102.88	41.95
6.99	1.40	34.60	14.23	107.32	43.70
7.71	1.69	38.81	15.94	111.80	45.36
8.43	2.02	43.86	17.96	116.32	47.08
9.34	2.43	49.18	20.10	120.89	48.68
10.42	2.94	54.07	22.08	125.51	50.48
11.48	3.45	58.68	23.93		
12.50	3.97	63.21	25.80		

PA					
2.77	0.18	83.24	34.08	230.24	81.30
2.97	0.18	87.56	35.84	235.19	82.56
3.47	0.28	91.95	37.63	240.07	83.87
4.17	0.44	96.44	39.44	244.91	85.08
4.97	0.67	100.94	41.19	249.80	86.28
5.84	0.96	105.46	42.94	254.74	87.66
6.82	1.33	110.00	44.71	259.63	88.87
7.86	1.76	114.57	46.42	264.58	90.17
8.90	2.23	119.10	48.07	255.46	87.54
10.08	2.78	123.59	49.67	261.90	89.20
11.31	3.37	128.14	51.28	266.53	90.57
12.48	3.96	132.75	52.85	271.41	91.97
13.77	4.60	137.42	54.48	276.34	93.37
15.18	5.30	142.07	56.00	281.31	94.77
16.69	6.08	146.71	57.52	286.34	96.01
18.34	6.93	151.42	59.03	291.31	97.49
21.02	8.22	156.14	60.46	296.24	98.88
23.94	9.61	163.18	62.66	301.22	100.28
26.66	10.85	168.93	64.32	306.24	101.59
29.79	12.21	172.40	65.38	311.21	103.02
33.07	13.60	177.21	66.79	316.24	104.26
36.76	15.11	181.95	68.22	321.31	105.76
41.08	16.86	186.73	69.56	326.33	107.12
46.36	18.99	191.55	70.92	331.39	109.19
51.76	21.18	196.32	72.23	336.47	111.28
56.61	23.12	201.12	73.60	341.50	113.18
61.22	24.98	205.98	74.90	346.56	114.76
65.65	26.79	210.77	76.20	351.68	116.09
70.08	28.64	215.62	77.48	356.75	117.35
74.53	30.45	220.51	78.70	361.88	118.58
78.91	32.31	225.35	80.03	367.07	119.75

PQ3					
204.46	74.50	267.39	90.91	322.93	106.77
208.76	75.64	272.36	92.29	325.90	107.75
213.62	76.98	277.28	93.59	328.85	108.57
218.54	78.24	282.25	94.95	331.78	109.61
223.39	79.58	287.27	96.42	334.69	110.75
228.20	80.83	292.24	97.83	337.59	111.74
233.05	82.08	297.25	99.25	340.46	112.94
237.96	83.35	302.31	100.72	343.38	114.00
242.82	84.66	307.32	102.19	346.36	114.62
247.73	85.80	311.26	103.31	350.50	115.66
252.70	87.10	314.15	104.19	355.70	116.70
257.61	88.34	317.02	104.99	360.94	118.23
262.47	89.63	319.94	106.01	366.33	119.50

PSC					
302.13	100.43	328.07	107.62	351.37	115.91
306.04	101.42	331.96	108.92	335.30	116.83
309.92	102.54	335.80	110.66	359.29	117.85
316.73	104.28	339.68	112.56	363.26	118.78
320.46	105.50	343.60	113.89	367.20	119.72
324.16	106.47	347.50	114.97		

Slow melt-crystallized					
X1					
313.68	100.48	334.31	105.03	354.66	109.02
319.20	101.78	339.45	105.98	359.76	109.98
324.23	103.02	344.55	107.03	364.93	111.03
329.24	104.00	349.62	107.95	370.06	112.10

XQ1					
154.40	60.16	219.90	79.19	288.89	95.25
157.77	61.24	224.72	80.49	293.97	96.24
162.51	62.70	229.62	81.73	299.01	97.33
167.18	64.18	234.57	82.92	304.00	98.39
171.89	65.56	239.46	84.12	308.97	99.45
176.65	67.03	244.31	85.38	313.99	100.54
181.34	68.38	249.22	86.47	319.08	101.67
186.08	69.82	254.19	87.61	324.23	102.85
190.86	71.18	259.11	88.73	329.35	103.97
195.59	72.56	264.00	89.81	334.42	104.99
200.36	73.87	268.95	90.94	339.57	105.97
205.21	75.23	273.97	91.99	344.67	107.03
210.09	76.68	278.95	93.11	349.74	107.94
215.02	77.98	283.89	94.14	354.78	108.95

Slow melt-crystallized					
XSC					
8.09	1.83	37.63	15.71	117.00	47.45
8.96	2.22	41.53	17.26	121.49	49.10
10.28	2.86	60.86	25.04	125.99	50.73
11.49	3.46	64.76	26.59	130.50	52.30
12.70	4.07	68.71	28.20	135.04	53.88
14.00	4.74	73.02	30.00	139.60	55.40
15.41	5.49	77.35	31.78	144.19	56.93
16.95	6.29	81.73	33.58	148.81	58.41
18.63	7.16	86.17	35.40	153.47	59.90
20.48	8.09	90.54	37.18	158.16	61.38
22.53	9.10	94.58	38.91	162.90	62.86
24.81	10.20	99.27	40.66	167.56	64.28
27.39	11.37	103.66	42.39	172.26	65.73
30.33	12.66	108.03	44.07		
33.72	14.11	112.52	45.78		

XQ2					
3.02	0.19	92.59	38.08	247.92	86.21
3.69	0.31	97.01	39.83	252.90	87.41
4.55	0.52	101.45	41.60	257.83	88.51
5.57	0.83	105.92	43.30	262.72	89.49
6.70	1.24	110.43	45.06	267.68	90.61
7.92	1.75	114.98	46.77	272.71	91.65
9.19	2.33	119.58	48.45	277.70	92.76
10.46	2.94	124.15	50.11	282.64	93.80
11.68	3.54	128.59	51.58	287.56	94.81
12.88	4.17	133.18	53.19	292.44	95.84
14.19	4.83	137.90	54.81	297.38	96.87
15.62	5.59	142.53	56.36	302.39	97.91
17.18	6.40	147.18	57.89	306.36	98.74
18.88	7.29	151.86	59.40	309.39	99.48
20.69	8.19	156.58	60.95	312.32	100.10
22.62	9.14	166.01	63.90	315.16	100.67
24.84	10.20	170.73	6538	318.06	101.41
27.40	11.37	175.49	66.77	320.95	102.29
30.23	12.61	180.18	68.22	323.82	102.73
33.43	13.98	184.93	69.58	326.68	103.38
37.24	15.55	189.72	70.98	329.54	103.95
41.71	17.32	194.45	72.30	332.45	104.55
47.01	19.44	199.23	73.65	335.44	105.12
52.32	21.56	204.07	74.96	338.41	105.79
57.09	23.51	208.85	76.27	341.37	106.44
61.76	25.41	213.68	77.59	347.49	107.42
66.26	27.24	218.57	78.96	350.99	108.23
70.64	29.04	223.41	80.17	354.89	108.97
74.95	30.82	228.31	81.40	358.77	109.75
79.32	32.68	233.26	82.66	362.63	110.52
83.76	34.47	238.16	83.84	366.06	111.22
88.18	36.25	243.01	85.16	369.93	112.08

Quenched film					
Fl					
9.95	2.72	12.25	3.83	14.86	5.12
11.00	3.22	13.50	4.45	16.34	5.84

F2					
190.74	70.46	200.22	73.07	209.90	75.73
195.51	71.80	205.02	74.40	214.74	77.02

F3					
4.79	0.62	109.73	44.52	275.89	93.98
5.72	0.93	114.20	46.18	280.78	95.65
6.86	1.36	118.75	47.86	285.75	97.17
8.09	1.87	123.29	49.50	290.68	98.75
9.36	2.44	127.82	51.09	295.57	100.46
10.63	3.03	132.46	52.71	299.45	101.93
11.84	3.61	137.11	54.26	302.34	103.34
13.06	4.21	141.76	55.73	304.94	104.92
14.37	4.84	146.43	57.29	307.25	106.62
15.80	5.56	151.12	58.72	309.65	107.40
17.36	6.32	155.84	60.26	312.05	108.33
19.06	7.15	165.25	63.10	314.45	109.72
20.92	8.03	169.91	64.51	316.92	110.87
22.99	9.01	174.64	65.86	319.37	112.15
27.86	11.19	179.32	67.26	321.83	113.52
30.57	12.34	184.07	68.62	324.27	114.73
33.64	13.64	188.91	69.98	326.71	116.28
37.30	15.15	193.69	71.29	329.14	117.82
41.61	16.87	198.41	72.61	331.58	119.69
46.77	18.98	203.10	73.86	334.02	122.34
51.99	21.10	207.86	75.16	336.51	122.43
56.65	23.01	212.71	76.51	338.94	122.52
61.09	24.81	217.51	77.78	341.29	122.99
65.52	26.66	222.27	79.09	343.69	123.22
69.98	28.49	227.11	80.43	346.16	123.88
74.38	30.34	232.04	81.73	348.69	123.51
78.73	32.18	236.93	82.97	351.19	124.13
83.08	34.00	241.77	84.21	353.60	124.84
87.44	35.74	246.56	85.90	355.99	124.99
91.82	37.53	251.44	86.94	358.37	125.34
96.24	39.29	256.41	88.31	360.73	125.86
100.71	41.06	261.34	89.69	363.60	126.71
105.24	42.89	266.23	91.15		

Quenched film					
FQ					
66.73	27.16	160.29	61.64	266.24	91.06
70.56	28.72	165.02	63.09	274.10	92.39
74.84	30.51	169.83	64.53	276.03	93.89
78.99	35.25	174.57	65.93	281.06	95.46
83.25	33.99	183.88	68.60	286.04	97.01
87.62	35.77	188.59	69.99	290.98	98.61
91.95	37.54	193.37	71.32	295.87	100.21
96.26	39.27	198.11	72.61	300.85	101.87
100.71	41.02	202.92	73.92	305.90	103.60
105.16	42.75	207.82	75.23	310.91	105.22
109.62	44.45	212.68	76.54	315.88	106.98
114.09	46.13	217.48	77.86	320.93	108.37
118.59	47.79	222.24	79.09	326.07	110.15
123.13	49.41	227.09	80.41	331.15	111.83
127.68	51.01	232.03	81.60	336.20	113.57
132.28	52.60	236.92	82.96	341.21	115.42
136.91	54.18	241.76	84.39	346.29	117.49
141.61	55.73	246.56	85.65	351.44	119.65
146.22	57.23	251.44	86.92	356.53	121.85
150.89	58.71	256.42	88.26	361.57	123.83
155.63	60.18	261.35	89.59	366.69	125.52

FSC					
267.79	90.94	301.99	101.85	337.06	112.99
272.40	92.70	307.03	103.54	342.05	114.98
277.34	94.01	312.03	105.09	347.12	117.55
282.23	95.54	316.99	106.74	352.35	120.70
287.20	97.03	322.04	108.22	357.66	122.66
292.13	98.71	327.04	109.75	362.79	124.53
297.02	100.22	332.01	111.30	367.89	125.98

FRT					
296.81	100.06	319.78	107.85	343.70	116.07
298.74	100.81	321.79	108.46	346.53	117.38
300.63	101.43	323.73	109.10	349.45	119.00
302.51	102.15	325.66	109.69	351.51	119.86
304.46	102.96	327.59	110.23	335.46	120.86
306.39	103.51	329.46	110.72	355.45	122.06
308.38	104.22	331.43	111.42	357.43	122.91
310.30	104.66	333.45	112.07	359.46	123.61
312.17	105.41	335.41	112.70	361.48	124.05
314.08	106.10	337.41	113.47	363.49	124.72
315.99	106.64	339.67	113.50		
317.89	107.17	341.77	114.87		

**Table 4:** Thermodynamic functions of crystalline PCTFE (units in J, K, and mol. 1 mol = 116.47 g)

*T* K	*C*_*p*_ J K^−1^ mol^−1^	*H* −*H*_0,_*_x_* J mol^−1^	*S* J K^−1^mol^−1^	−(*G* −*H*_0_*_,x_* J mol^−1^
5	0.65	0.92	0.25	0.33
10	2.71	8.96	1.28	3.88
15	5.26	28.83	2.86	14.07
20	7.84	61.64	4.73	32.97
30	12.43	163.95	8.82	100.50
40	16.46	308.19	12.94	209.26
50	20.48	492.90	17.04	359.17
60	24.51	717.79	21.13	550.03
70	28.60	983.28	25.22	781.76
80	32.72	1289.8	29.30	1054.3
90	36.82	1637.6	33.39	1367.8
100	40.84	2026.0	37.48	1722.2
120	48.43	2919.9	45.61	2553.3
140	55.51	3961.8	53.63	3546.0
160	61.96	5137.2	61.47	4697.2
1SO	68.05	6437.9	69.12	6003.3
200	73.82	7857.2	76.59	7460.7
220	79.24	9388.4	83.88	9065.7
240	84.27	11024	91.00	10815
260	88.91	12757	97.93	12704
273.15	91.78	13945	102.72	14113
280	93.31	14579	105.01	14825
298.15	97.22	16310	111.00	16785
300	97.64	16489	111.60	16991
320	101.81	18485	118.04	19288
340	105.79	20561	124.33	21712
360	109.61	22716	130.49	24260
380	113.24	24945	136.52	26931
400	116.71	27246	142.41	29720
420	119.99	29613	148.19	32626
440	123.11	32045	153.85	35647
460	126.05	34537	159.38	38780
480	128.81	37087	164.81	42022
497	131.03	39262	169.33	44895

**Table 5:** Thermodynamic functions of amorphous and liquidus PCTFE (Units in J, K, and mol. 1 mol = 116.47 *g*)

*T* K	*C*_*p*_ J K^−1^ mol^−1^	*H*−*H*_0,gl_ J mol^−1^	*S*−*S*_0,gl_ JK^−1^ mol^−1^
5	0.69	0.95	0.26
10	2.74	9.20	1.32
15	5.16	28.90	2.88
20	7.59	60.82	4.70
30	12.09	159.65	8.65
40	16.24	301.47	12.70
50	20.30	484.16	16.60
60	24.39	707.57	20.82
70	28.54	972.18	24.89
80	32.71	1278.4	28.98
90	36.83	1626.2	33.07
100	40.84	2014.7	37.16
120	48.39	2908.0	45.28
140	55.04	3943.5	53.25
160	61.34	5107.8	61.01
180	67.37	6395.3	68.59
200	73.14	7800.9	75.99
220	78.65	9319.2	83.22
240	83.90	10945	90.29
260	88.89	12673	97.20
273.15	93.00	13869	101.68
280	95.59	14514	104.02
298.15	103.93	16323	110.27
300	104.90	16515	110.92
320	116.81	18729	118.06
335	127.43	20559	123.65
340	127.97	21198	125.54
360	130.04	23778	132.91
380	131.98	26399	139.99
400	133.79	29056	146.81
420	135.46	31749	153.38
440	137.01	34474	159.72
460	138.42	37229	165.84
480	139.70	40010	171.76
497	140.68	42393	176.64
500	140.84	42816	177.48
520	141.86	45643	183.03
540	142.74	48489	188.40
560	143.49	51352	193.60
580	144.10	54228	198.65
600	144.59	57115	203.54
620	144.94	60010	208.29

## Footnotes

2 Certain commercial equipment, instruments, or materials are identified in this paper to specify adequately the experimental procedure. Such identification does not imply recommendation or endorsement by the National Institute of Standards and Technology, nor does it imply that the materials or equipment identified are necessarily the best available for the purpose.
